# Response of Parathyroid Hormone to Vitamin D Deficiency in Otherwise Healthy Individuals

**DOI:** 10.7759/cureus.9764

**Published:** 2020-08-15

**Authors:** Mayra Z Malik, Omar B Latiwesh, Fatimah Nouh, Azhar Hussain, Sohail Kumar, Jasndeep Kaler

**Affiliations:** 1 Internal Medicine, Combined Military Hospital Lahore Medical College and Institute of Dentistry, New York, USA; 2 Pathology, Higher Institute of Medical Professions, Benghazi, LBY; 3 Biochemistry, Higher Institute of Medical Professions, Benghazi, LBY; 4 Biochemistry, Faculty of Medicine, University of Benghazi, Benghazi, LBY; 5 Healthcare Administration, Franklin University, Columbus, USA; 6 Medicine, Xavier University School of Medicine, Oranjestad, ABW; 7 Internal Medicine, Dow Medical College, Dr. Ruth K. M. Pfau Civil Hospital, Karachi, PAK

**Keywords:** vitamin d, pth, secondary hyperparathyroidism, vitamin d deficiency

## Abstract

Background and objectives

Vitamin D deficiency is a global public health issue, which affects people of all ages and ethnicities. However, severe deficiency seems to be more prevalent in the Middle East and South Asia. Evidence suggests that low serum 25-hydroxycholicalciferol [25(OH)D] levels are associated with an increase in parathyroid hormone (PTH). Yet, the 25-OHD levels leading to serum PTH increase are still a matter of debate. The objective of this study is to assess deficiency of vitamin D in otherwise healthy individuals, and to determine the response of the PTH to vitamin D deficiency.

Methods

This observational study was conducted from January 2018 to May 2018. A total of 43 individuals were selected from three separate clinics in Libya (Alrazy clinic, Alhaya clinic, and Alnukbah clinic). Blood drawn from these individuals was assessed for serum calcium, phosphorus, 25(OH)D, and PTH. These data were collected and analyzed using the Statistical Package for Social Sciences (SPSS) version 17.0 for Windows (SPSS Inc., Chicago, IL).

Results

The mean age and standard (SD) of the study participants was 47.4 ± 12.4. The age range was 19-67 years. The ratio of male to female was 1:2. The percentage of individuals with vitamin D deficiency in the study group was 95.3%, whereas the percentage of vitamin D insufficiency was 4.7%. These data suggest that individuals with severe deficiency show higher PTH values (75.66 ng/ml), whereas those with insufficiency showed lower PTH values (37.5 ng/ml).

Conclusion

The population in the present study was overall deficient in 25-OH vitamin D, which indicates a greater need for supplementation with vitamin D. However, not all the individuals with vitamin D deficiency have high levels of PTH, a finding that agrees with the need for new criteria in the management of vitamin D deficiency and the importance of PTH testing.

## Introduction

Vitamin D is a fat-soluble vitamin that is responsible for the absorption of calcium, magnesium, and phosphate in the proximal and distal intestine via vitamin D receptors. Although vitamin D can be directly taken from food sources, such as dairy products, fishes, and oils, a major portion of the body’s vitamin D is synthesized de novo via exposure of skin to sunlight (UVB rays), resulting in cholecalciferol (vitamin D3), which is further converted to 25-hydroxycholecalciferol [25(OH)D], in the liver. Vitamin D status is best determined by measuring serum 25(OH)D. Although inactive, it is the major circulating form. Further hydroxylation of 25(OH)D in the kidneys yields calcitriol or 1,25 dihydroxycholecalciferol, the biologically active form.

Evidence has reported that the function of vitamin D is not limited to maintenance of calcium homeostasis, but it also maintains various cellular processes with its effects on multiple systems [1]. Vitamin D deficiency (VDD) can be caused by a multitude of factors, such as decreased sun exposure, nutritional deficits, gastrointestinal diseases such as celiac disease and gastric surgery, and the chronic use of drugs such as anticonvulsants and corticosteroids [2]. Decreased 25(OH)D leads to decreased calcium absorption, and lowered serum calcium levels trigger the release of parathyroid hormone (PTH). This rise in PTH attempts to maintain calcium homeostasis at the cost of increased bone turnover, resulting in bone loss. This bone loss combined with mineralization deficits due to decreased calcium absorption contributes to an increased risk of fractures, particularly in the geriatric population. There is still uncertainty about what levels of 25(OH)D should be considered normal and abnormal, due to different methods used by different laboratories to assess vitamin D levels. A research carried out in Saudi Arabia observed that amongst the patients with VDD, PTH was raised when measured with high-performance liquid chromatography-liquid chromatography with mass spectrometry (HPLC-LC.MS) and normal when measured with chemiluminescence immunoassay (CLIA) [3]. Hence, a discrepancy lies in the measurement of deficiency and insufficiency of 25(OH)D through different assays.

Secondary hyperparathyroidism occurs as the body’s response to low levels of calcium due to hypovitaminosis D (VDD). Supplementation with calcidiol has shown to raise serum 25(OH)D, substantially lower PTH levels, and reduce the incidence of secondary hyperparathyroidism. However, the study of Sahota et al. showed that half the patients with VDD did not have an adequate PTH response [4]. Hence, the level of 25(OH)D sufficient enough to prompt a rise in the PTH is still not known with certainty. The differences in findings prompted the need for this study, which is the first of its kind to be conducted in Libya. Our study aims to assess the levels of vitamin D in healthy individuals of Libya and to determine the response of PTH to the levels of vitamin D.

## Materials and methods

The present observational study was conducted between January 2018 and May 2018. A total of 43 clinically healthy individuals, with ages between 19 and 67 years, were selected from three different clinics in Libya (Alrazy clinic, Alhaya clinic, and Alnukbah clinic). The first 43 patients with a habitually low intake of vitamin D were included in the study. Subjects with a current or past history of the following were excluded from the study: diabetes mellitus, malabsorption, chronic kidney disease, chronic diarrhea, infection, primary hyperparathyroidism, hypoparathyroidism, chronic liver disease, acute or chronic inflammation, tuberculosis, and malignancy. Women who were pregnant and lactating mothers were also excluded. Informed consent was received from all the participants after being fully aware of the research goals and protocols. This study was regulated by the Ethical Board of Benghazi Medical Center, the date of registration was November 13, 2017, and the registration ID number is 5131117.

Sample collection

A non-fasting 3-5 ml of the blood sample was obtained from each participant in the morning via routine venipuncture for determining serum levels of 25(OH)D, calcium (Ca), phosphorous (P), and PTH. The serum was collected in a plain tube, allowed to clot, and then centrifuged. The serum was stored at -20°C until the analysis was done. Serum 25(OH)D concentrations were estimated by the microplate washer and reader (LINEAR, Barcelona, Spain) using a commercially available ELISA kit (Euroimmun, Lübeck, Germany). Serum Ca and P were analyzed by the COBAS INTEGRA 400 plus analyzer (Roche Ltd, Basel, Switzerland) using COBAS packs (Roche Ltd, Basel, Switzerland) as per the manufacturer's protocol. The serum PTH was measured by an electrochemiluminescent assay using the COBAS e411 (Roche Diagnostics GmbH, Mannheim, Germany).

Statistical analysis

Serum PTH levels between the ranges 30 and 65 pg/ml were considered normal. PTH levels above or below these ranges were classified as being high and low, respectively. Circulating 25(OH)D levels were classified into three groups according to the following criteria: vitamin D insufficiency, 25(OH)D level of 21-29 ng/ml; mild VDD, 25(OH)D level of 10-20 ng/ml, and severe VDD, 25(OH)D level of <10 ng/ml.

Statistical Package for Social Sciences (SPSS) version 17.0 for Windows (SPSS Inc., Chicago, IL).was used to analyze the data. The results of the study were expressed as mean and standard deviation (SD). P-value of ≤0.05 (5%) was considered statistically significant.

## Results

The mean age of the participants was 47.4 ± 12.4 years. The results were obtained from a female dominant population with a male to female ratio of 1:2. The mean serum levels of vitamin D, PTH, calcium, and phosphorous are shown in Table 1.

**Table 1:** Mean ± SD values of serum levels of vitamin D, parathyroid hormone, calcium, and phosphorus (n = 43). PTH, parathyroid hormone; SD, standard deviation

Variables	Mean ± SD	Range
Vitamin D (ng/ml)	11.29 ± 5.04	25-80
PTH (pg/ml)	63.32 ± 33.31	30-65
Calcium (mg/dl)	9.16 ± 0.43	8.5-10.5
Phosphorus (mg/dl)	4.60 ± 5.85	2.7-4.7

**Figure 1:**
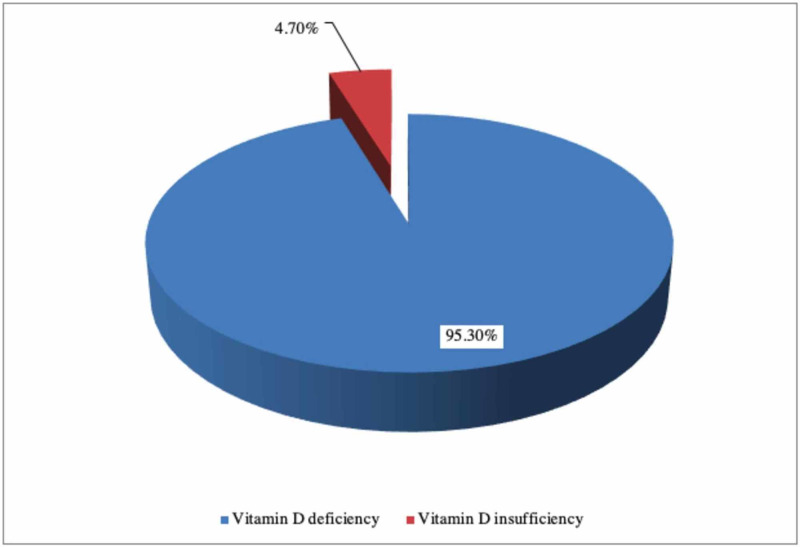
Percentage of vitamin D deficiency in the study group.

As shown in Figure 1, the majority of the study population had VDD accounting for about 95.3% (n = 41), whereas the remaining 4.7% (n = 2) of the population had vitamin D insufficiency. Taking the severity of VDD into account, 48.8% (n = 21) of the entire study population had a severe deficiency and 46.5% (n = 20) had mild VDD as shown in Table 2.

**Table 2:** Classification of the entire sample on the basis of serum vitamin D levels (n = 43).

Criteria	Frequency (n)	Percentage (%)
Severe vitamin D deficiency (<10 ng/ml)	21	48.8
Mild vitamin D deficiency (<10 ng/ml)	20	46.5
Vitamin D insufficiency (21-29 ng/ml)	2	4.70

**Figure 2:**
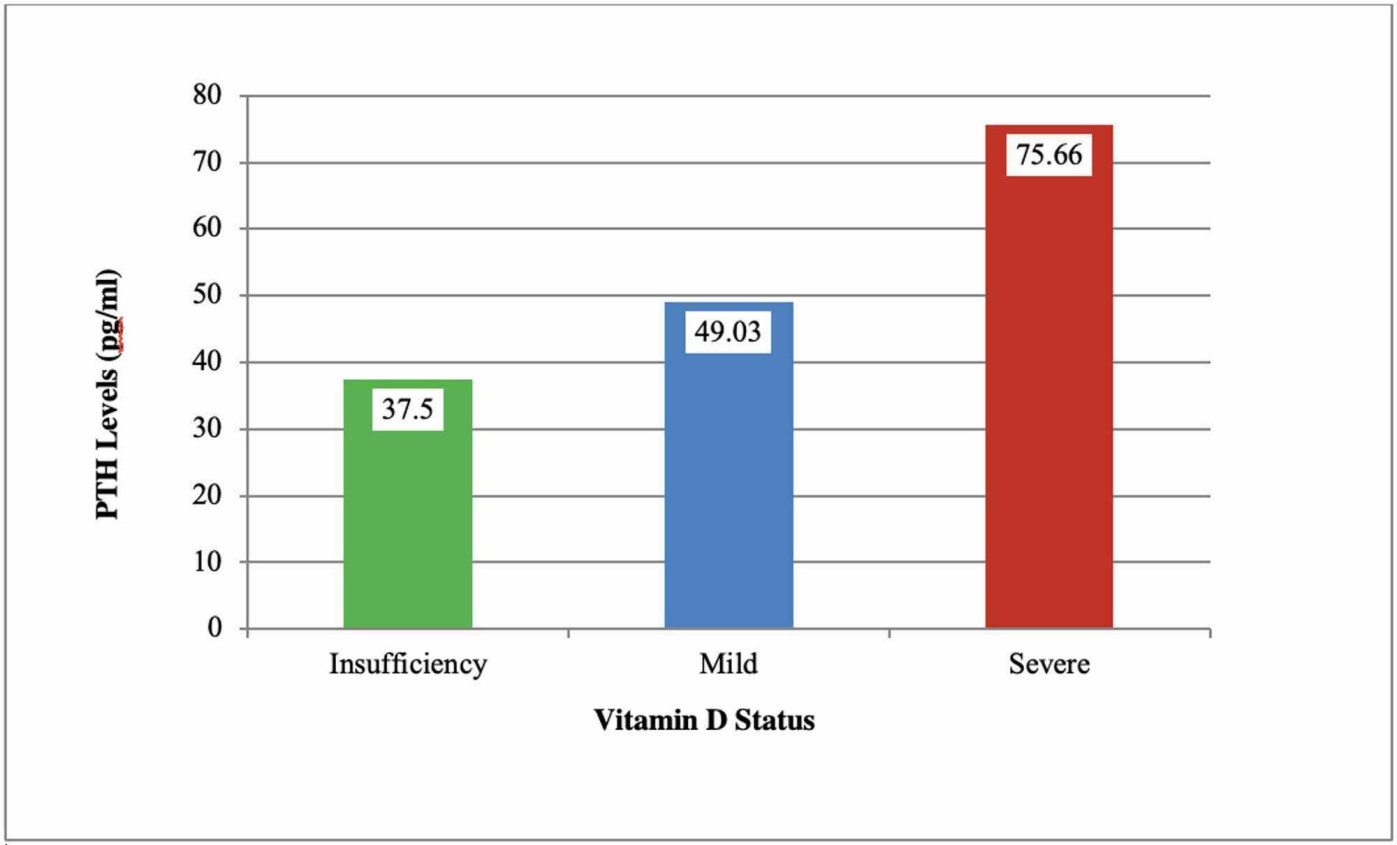
Parathyroid hormone (PTH) levels according to vitamin D deficiency categories in the study group.

Figure 2 and Table 3 demonstrate the relationship between vitamin D and the levels of PTH. The results showed that while more than half (61.1%) of the patients suffering from severe VDD had serum PTH levels above the normal range, nearly three-fourth (72.7%) of the patients with mild deficiency of vitamin D had serum levels of PTH within the normal range. Moreover, all (100%) patients with vitamin D insufficiency also reported normal serum PTH levels.

**Table 3:** Relationship between vitamin D status and serum levels of parathyroid hormone (PTH).

Criteria	Low PTH (<35 pg/ml)	Normal PTH (30-65 pg/ml)	High PTH (>60 pg/ml)
	n (%)	n (%)	n (%)
Severe vitamin D deficiency (n = 21)	0 (0)	8 (38.9)	13 (61.1)
Mild vitamin D deficiency (n = 20)	2 (9.1)	14 (72.7)	4 (18.2)
Vitamin D insufficiency (n = 2)	0 (0)	100 (100)	0 (0)

Furthermore, laboratory data revealed the highest mean serum levels of PTH and the lowest levels of Ca and P among patients with severe vitamin deficiency. In contrast, those with insufficiency and mild deficiency of vitamin D showed lower serum levels of PTH. Table 4 demonstrates mean serum levels of PTH, Ca, and P in relation to vitamin D deficiency and insufficiency. Thus, our results were highly suggestive of the negative relationship between 25(OH)D and PTH.

**Table 4:** Mean serum levels of parathyroid hormone (PTH), calcium, and phosphorus in relation to vitamin D deficiency and insufficiency.

Criteria	PTH (pg/ml)	Calcium (mg/dl)	Phosphorus (mg/dl)
Severe vitamin D deficiency	75.66	9.05	4.50
Mild vitamin D deficiency	49.03	9.19	4.94
Vitamin D insufficiency	37.50	9.50	4.65

## Discussion

VDD is currently a significant nutritional and health concern around the world. It is a multifactorial condition, influenced by weight, age, clothes, the color of skin, geographical location, atmospheric condition, use of sunscreen (that prevents the ultraviolet B rays from penetrating the skin), the incidence of various chronic illnesses, and a number of drugs. Reduced dietary intake may be another cause as most foods are not fortified with vitamin D [2,5]. Modern lifestyle habits that limit outdoor activity may also be to blame. The optimum level of 25(OH)D required for the maintenance of bones and muscles is still a controversy. However, according to the Endocrine Society, 25(OH)D levels <29 ng/ml are deemed insufficient [6].

Over the last decade, scientific communities have been keenly studying the role of vitamin D in influencing the health of the general population. This is attributed to the deeper understanding of its significance not only in bone metabolism but also in various cell signaling pathways and a multitude of organ systems, such as the immune, vascular, and nervous systems. Moreover, deficiency of vitamin D has been linked to increased risk of diabetes, obesity, cardiovascular, respiratory, autoimmune illnesses, and cancer [1]. The major consequence of hypovitaminosis D is bone remodeling, which correlates to PTH levels. Our study assesses vitamin D levels in otherwise healthy individuals and the response of PTH to its deficiency.

Our study demonstrated that a high percentage (95.3%) of apparently healthy Libyan individuals had VDD. Despite being one of the sunniest parts of the world, the vitamin D problem in Benghazi is similar to parts of Asia, Africa, and the Middle East [7]. In Egypt, vitamin D deficiency and insufficiency are reported to be 77% and 15%, respectively, with 9% of the population having sufficient levels of vitamin D [8]. In Qatar 83%-91% of the population and in Tunisia, 47.6% of the population are vitamin D deficient [9,10]. In Saudi Arabia, deficiency of vitamin D has reached 67.8% [11]. In a recent systematic analysis, which included 195 reports from 44 countries around the world using the same cut-off values used in the present study, the mean value of 25(OH)D was <30 ng/ml in 88.1% of the reported samples and <20 ng/ml in 37.3% of the samples [12].

In a study carried out on the normal healthy population of Saudi Arabia, it was found that PTH was increased in 51.8%, 66.23%, and 100% of patients with VDD as measured by CLIA, radioimmunoassay (RIA), and HPLC-LC.MS, respectively [3]. Kilicarslan et al. in their study reported that more than 75% of patients with VDD had normal PTH levels [13]. The results of these studies are more or less similar to our study in which high levels of PTH have been observed mostly in individuals with severe hypovitaminosis D. Interestingly, about 72% of participants with mild deficiency of vitamin D had normal PTH levels. All the participants with vitamin D insufficiency had normal PTH values. In a study undertaken by Elsammak et al., in a limited number of patients, levels of PTH did not correlate with the levels of serum 25(OH)D in either of the genders [14].

Several studies conducted on a healthy population observed that serum PTH is inversely related to 25(OH)D [15,16]. More than 70 studies have reported fluctuating levels of 25(OH)D between 10 and 50 ng/ml, without any clear-cut value that leads to the decline in PTH level [17]. A majority of the studies show that the concentrations of serum PTH begin to decline when levels of 25(OH)D increase to 15-20 ng/ml and are maximally suppressed at 30-40 ng/ml (75-100 nmol/L) [18-20]. Some evidence suggests that factors such as age and ethnicity may influence the relation between 25(OH)D and PTH [21,22]. Our study also demonstrates a similar inverse relationship between severely decreased levels of vitamin D and PTH. However, this relation seems to end as vitamin D levels enter mildly deficient mid-range. Another research, utilizing the average thresholds of 25(OH)D from a very broad dataset, showed that there is no asymptote because the fluctuating vitamin D-dependent PTH levels did not reveal any cut-off point beyond which 25(OH)D did not further suppress PTH [21].

According to the physiological paradigm, deficiency of vitamin D results in increased secretion of PTH to maintain homeostasis of calcium, which occurs at the expense of increased bone turnover [23]. However, some vitamin D-deficient individuals do not exhibit increased PTH secretion, and this condition has been termed as ‘functional hypoparathyroidism’ [4]. There are other factors that may influence the body’s normal PTH response. These factors may vary between the two genders and include IGF-1 and testosterone in men, and BMI, smoking, kidney function, and magnesium levels in women. Thus, these factors should be taken into account when describing adequate 25(OH)D status from PTH levels [24].

Regardless of the status of PTH levels, the Endocrine Society Clinical Practice Guideline has recommended vitamin D2 or D3 (25(OH)D) for treatment or prevention of VDD [6]. The study of Sahota et al. and another study of Lips found that the combination of calcium and vitamin D supplementation may be effective in the prevention of hip and other peripheral fractures in patients with VDD accompanied by secondary hyperparathyroidism, but its role in patients with VDD without secondary hypoparathyroidism and vitamin D-replete subjects needs further evaluation [4,25]. Hence, according to these studies, calcium supplementation is required when there is hyperparathyroidism secondary to VDD, as calcium homeostasis is maintained at the expense of increased bone turnover.

Limitations

The findings of this study should be seen in the light of a few limitations. In regards to the baseline subject characteristics, due to the small sample size, there may have been larger than anticipated disparities in gender and age. A large sample size would help ensure an equal representation of both genders and a constant age range. Excluding those with an abnormal BMI would also aid this study, since obesity may also play a role in VDD. Enquiring the subjects of additional factors that may impact serum vitamin D levels including hours of daytime exposure to sunlight, use of sunscreen, vitamin D and calcium intake in the diet, as well as the intake of any vitamins or multivitamins prior to the sampling.

## Conclusions

We conclude that the population in the present study was overall deficient in 25-OHD, indicating a greater need for vitamin D supplementation. Although we observed an inverse relationship between vitamin D and PTH in individuals with severe 25(OH)D deficiency, most of the individuals having vitamin D in the mid-range and those that have vitamin D insufficiency have normal PTH levels. This implies that the current criteria and guidelines are unsuitable, and there is a dire need for revised criteria on the management of VDD. Our study also highlights the importance of PTH testing in regard to VDD. In order to further validate the results of our research, more studies are required in Libya and other geographical regions.
